# Diversity of culturable bacterial endophytes of saffron in Kashmir, India

**DOI:** 10.1186/s40064-015-1435-3

**Published:** 2015-11-02

**Authors:** Tanwi Sharma, Sanjana Kaul, Manoj K. Dhar

**Affiliations:** School of Biotechnology, University of Jammu, Jammu, 180006 India

**Keywords:** Endophytic bacteria, Bioactive, BIOLOG, Saffron, Biological stress, Diversity

## Abstract

Saffron (*Crocus sativus*) is a medicinally important plant. The Kashmir valley (J&K, India) emblematizes one of the major and quality saffron producing areas in the world. Nonetheless, the area has been experiencing a declining trend in the production of saffron during the last decade. Poor disease management is one of the major reasons for declining saffron production in the area. Endophytes are known to offer control against many diseases of host plant. During the present study, culturable bacterial endophytes were isolated from saffron plant, identified and assessed for plant growth promoting activities. Molecular and phylogenetic analysis grouped the fifty-four bacterial isolates into eleven different taxa, viz. *Bacillus licheniformis*, *B. subtilis*, *B. cereus*, *B. humi*, *B. pumilus*, *Paenibacillus elgii*, *B. safensis*, *Brevibacillus* sp., *Pseudomonas putida*, *Staphylococcus hominis* and *Enterobacter cloacae*. The results were also supported with the identification based on BIOLOG system. *B. licheniformis* was the dominant endophyte in both leaves and corms of saffron. 81 % isolates showed lipase activity, 57 % cellulase, 48 % protease, 38 % amylase, 33 % chitinase and 29 % showed pectinase activity. 24 % of the isolates were phosphate solublizers, 86 % showed siderophore production and 80 % phytohormone production potential. The present repository of well characterized bacterial endophytes of saffron, have plant growth promoting potential which can be explored further for their respective roles in the biology of the saffron plant.

**Electronic supplementary material:**

The online version of this article (doi:10.1186/s40064-015-1435-3) contains supplementary material, which is available to authorized users.

## Background

Saffron, commonly called as Zafran, Kesar, Kong, Kong Posh etc. and scientifically known as *Crocus sativus* belongs to the family Iridaceae of the order asparagales (Katariya et al. 2011). It is a small perennial plant with purple colored flowers. The flower stalk and leaves rise from corm, which is an underground part of the plant (Bhargava 2011). Apart from its uses as flavouring and colouring agent, the spice also possesses several medicinal properties (Katariya et al. 2011). Traditionally, stigmas of the plant are used for the treatment of different human disorders. Some of the reported pharmacological properties of the various solvent extracts of *Crocus sativus* include: anticancer (Singla and Bhat 2011; Bakshi et al. 2010); antinociceptive and anti-inflammatory (Hosseinzadeh and Younesi 2002); anticonvulsant and antidepressant (Akhondzadeh et al. 2005; Dharmananda 2005); antioxidant (Papandreou et al. 2006); hypolipidemic (Asdaq and Inamdar 2010); antityrosinase (Sariri et al. 2011); immunomodulatory (Kianbakht and Ghazavi 2011) and many other bioactivities (Bhargava 2011; Katariya et al. 2011).

Jammu and Kashmir is the only state in India where saffron is grown. In Kashmir, Pampore town is famous worldwide for its high grade saffron (Kozgar and Jabeen 2012). Unfortunately, total area under saffron cultivation in Kashmir has declined from 5707 ha in 1997 to 2667 ha in 2007 whereas the yield has observed a dip from 15.95 to 5.61 tonnes in the decade (Husaini et al. 2013). Biological stress caused by various pathogens is one of the main reasons for the declining trend in saffron yield. Some of the common fungal pathogens which infect the saffron and cause corm rot are *Rhizoctonia crocorum*, *Phoma crocoplila*, *Fusarium moniliforme*, *Macrophomina phaseolina*, *Fusarium oxysporum*, *f.* sp. *solani*, *F. pallidoroseum*, *F. equiseti*, *Mucor* sp., *Penicillium sp.* and *Sclerotium rolfsii* (Husaini et al. 2010). Among these pathogens, *Fusarium oxysporum f. solani* and *Fusarium oxysporum f.* sp. *gladioli* are the most devastating in Kashmir raising the corm rot incidence rate to 70–85 % leading to huge loss in terms of crop yield (Kalha et al. 2007). *Fusarium* corm rot of saffron caused by *F.oxysporum f.* sp. *gladioli* is also reported to cause huge yield losses in Italy as well (Primo et al. 2002).

Plants host useful bacteria as endophytes, epiphytes and rhizobacteria. Bacterial endophytes dwell host plant tissues in a symptomless manner and therefore, function as plant growth promoters (PGPs). Plants are associated with plethora of such significant microbes having biocontrol, stress resistance, phytoremediation and growth promoting potential (Ryan et al. 2008). Bacterial genera viz. *Bacillus* and *Pseudomonas* have been reported to be extensively used as biocontrol agents against root rot diseases, as well as plant growth promoters of different crop plants (Preston 2004; Dasgupta et al. 2006; Gheorghe et al. 2008).

Diversity studies are important to understand the ecological role of endophytic microbes in the host plant system. In addition to genetic and taxonomic diversity, metabolic diversity is also important to determine the exact functional role of such microbes. Carbon source utilization is widely used to determine the functional diversity of bacterial communities of a specific environment. Knowledge about the endophytic fungal diversity of saffron is limited to few reports (Raj et al. 2013) whereas the endophytic bacterial diversity of saffron is still not known. In order to fill this lacunae, the present study was undertaken to explore the bacterial inhabitants of saffron plant endosphere and determine their putative role in the biosystem of the plant.

## Results and discussion

During the present study, a total of fifty-four bacterial endophytes were isolated from 252 surface sterilized leaf and corm segments of healthy saffron plants. Screening based on morphological and biochemical features broadly classified the isolates into 21 different morphospecies (Table 1; Additional file 1: Tables S1 and S2) which include fifteen phenotypically different endophytic bacterial isolates from leaves of saffron and six isolates from the corm. Isolation rate (IR) values represent percent number of endophytes isolated with respect to total number of tissue segments incubated. Isolation rate of bacterial endophytes in leaf tissue was found to be higher as compared to those in corm tissue (Table 2), which might be attributed to the variation of endophytic population with the growth stages of the plant (Zinniel et al. 2002).

**Table 1:** Comparative analysis of endophytic bacterial isolates of saffron with respect to different parameters

Saffron plant part used	Endophytic bacterial isolate accession no.	Morphology (gram’s reaction)	BIOLOG Identification (similarity index value)	Molecular identification (%probability)	Genbank accession no.	Metabolic activities
Leaf	TS-2	Bacilli (+)	*Bacillus pumilus* (0.689)	*Bacillus pumilus* (100)	KM657271	Lip, amy, cel, pec, pro. Ph, ch, sd, I+
	TS-3	Bacilli (+)	*Brevibacillus borstelensis* (0.613)	*Brevibacillus* sp. (99)	KR780748	Lip, I+
	TS-4	Bacilli (+)	*Bacillus licheniformis* (0.802)	*Bacillus licheniformis* (100)	KM609050	Amy,sd, I+
	TS-5	Bacilli (+)	*Bacillus* sp. (0.687)	*Bacillus subtilis* (99)	KM609051	Lip, amy, pec, pro. Ch,sd, I+
	TS-6	Bacilli (+)	*Bacillus cereus* (0.574)	*Bacillus cereus* (98)	KM609052	Cel, sd, I+
	TS-7	Bacilli (+)	*Bacillus licheniformis* (0.620)	*Bacillus licheniformis* (99)	KM609053	Lip, amy, pro. Ph, sd, I+
	TS-8	Bacilli (ND)	*Bacillus pumilus* (0.679)	*Bacillus pumilus* (100)	KR528376	Lip, cel, sd
	TS-9	Bacilli (+)	ND	*Bacillus humi* (99)	KM657260	Cel, sd, I+
	TS-10	Bacilli (+)	*Bacillus pumilus* (0.689)	*Bacillus pumilus* (100)	KM657261	Lip, cel, sd, I+
	TS-11	Bacilli (+)	*Bacillus licheniformis* (0.549)	*Bacillus licheniformis* (100)	KR780747	Lip, I+
	TS-12	Bacilli (+)	*Bacillus cereus* (0.647)	*Bacillus cereus* (100)	KM657262	Lip, sd,
	TS-13	Bacilli (+)	*Bacillus subtilis ss subtilis* (0.578)	*Bacillus subtilis* (98)	KM657263	Lip, ch, sd, I+
	TS-14	Bacilli (+)	ND	*Pseudomonas putida* (99)	KR528377	Lip, cel, pec, pro Ch, sd, I+
	TS-15	Coccobacilli (ND)	ND	*Paenibacillus elgii* (98)	KM657264	Lip, amy, cel, pro Ph, ch, sd, I+
	TS-16	Bacilli (+)	*Bacillus licheniformis* (0.634)	*Bacillus licheniformis* (99)	KR858304	Lip, amy, cel, pec, pro Ch, sd, I+
Corm	TS-17	Bacilli (+)	*Bacillus licheniformis* (0.684)	*Bacillus licheniformis* (99)	KM657265	Lip, amy, pro Ph, ch, sd, I+
	TS-18	Bacilli (+)	ND	*Bacillus licheniformis* (98)	KM657266	Amy
	TS-20	Bacilli (+)	*Bacillus pumilus* (0.683)	*Bacillus pumilus* (99)	KM657267	Lip, cel, pec, pro, sd, I+
	TS-22	Bacilli (+)	*Bacillus safensis* (<0.500)	*Bacillus safensis* (100)	KM657268	Lip, cel, sd, I+
	TS-26	Cocci (+)	*Staphylococcus hominis ss hominis* (0.624)	*Staphylococcus hominis* (99)	KM657269	Lip, cel, pec, pro, sd, I+
	TS-27	Bacilli (−)	*Enterobacter cloacae* (0.529)	*Enterobacter cloacae* (100)	KM657270	Lip, cel, pro Ph, sd, I+

*ND* not determined, *lip, amy, cel, pec, pro, chi, sd, ph and i+* production of lipase, amylase, cellulase, pectinase, protease, chitinase, siderophore production, phosphate solubilisation and indole acetic acid production by the endophytic bacterial isolates of saffron

‘+’ Indicates bacterial isolate showing positive test for the substrate whereas ‘−’ sign indicates negative test

**Table 2:** Isolation rates of endophytic bacterial isolates of saffron (*C. sativus*)

	Corm	Leaf	Saffron plant (corm + leaf)
No. of explants	126	126	252
No. of isolates	13	41	54
Isolation rate (%)	10.32	32.54	21.43

16S rDNA sequences of all the isolates showed 99–100 % similarity with already available sequences in NCBI. On this basis twenty-one different morphotypes selected from fifty-four isolates were classified into eleven different bacterial species viz. *Bacillus pumilus*, *B. licheniformis*, *B. subtilis*, *B. cereus*, *B. humi*, *Brevibacillus* sp., *Pseudomonas putida*, *Paenibacillus elgii*, *Bacillus. safensis*, *Staphylococcus*. hominis and *Enterobacter*. cloacae (Table 1).

**Fig. 1:**
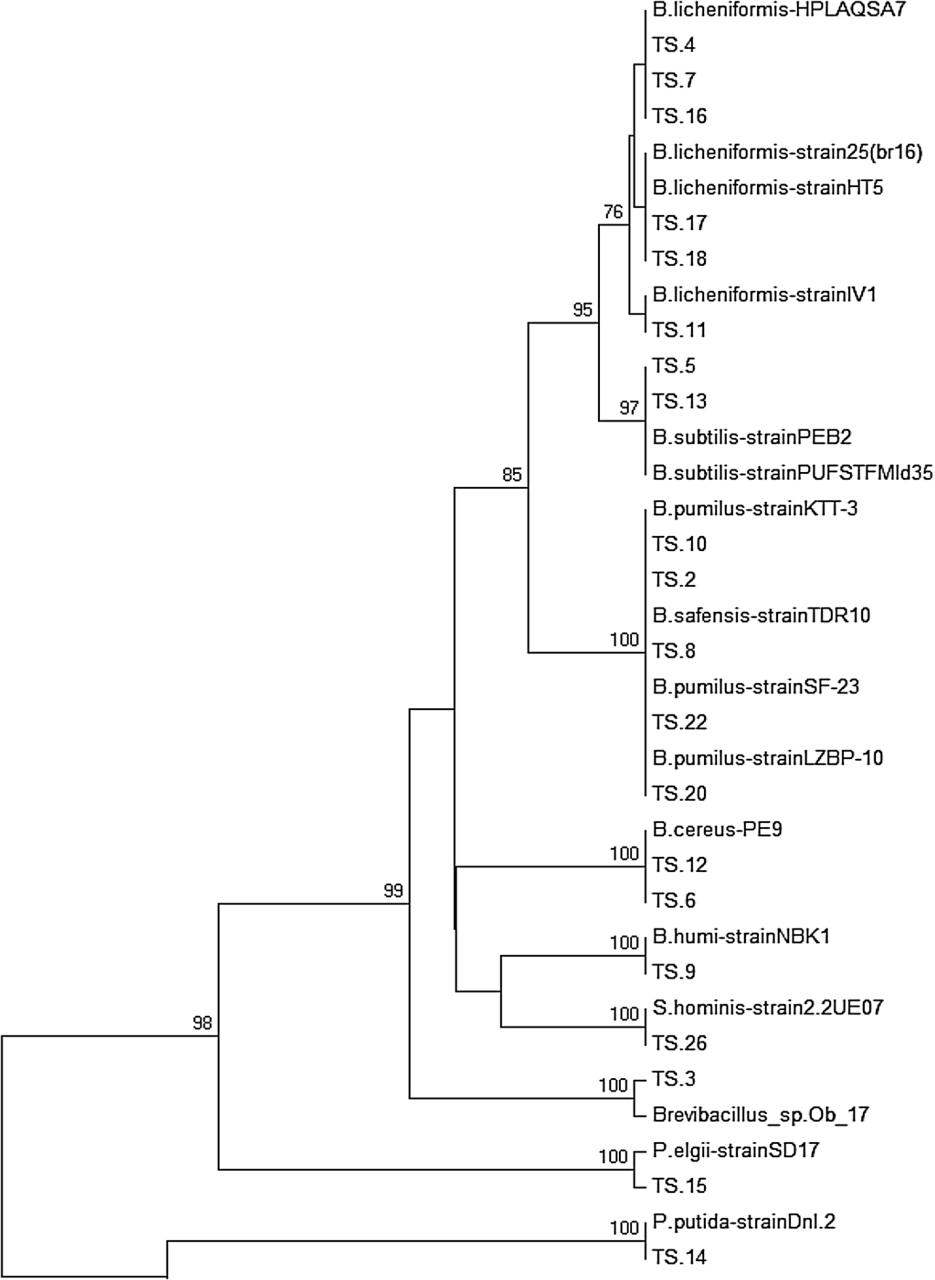
Evolutionary relationship among taxa of the endophytic bacterial isolates and with the reference taxa

Phylogenetic analysis divided the isolates into two main groups namely Firmicutes and Proteobacteria. The optimal tree with the sum of branch length 1.17970862 is shown in Fig. 1. The percentage of replicate trees in which the associated taxa clustered together in the bootstrap test (500 replicates) were shown above the branches (Fig. 1) (Felsenstein 1985).

**Fig. 2:**
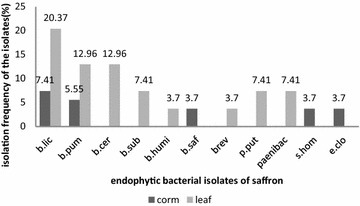
Isolation frequency (% values shown on *bars*) of bacterial endophytes from corm and leaf tissue of saffron. on X axis *B.lic, Bacillus licheniformis*; *B.pum, B. pumilus*, *B.saf*, *B. safensis*; *B.bei*, *B. beijingensis*; *B.cer*, *B. cereus*; *B.sub*, *B. subtilis*; *B.hum*, *B. humi*; *Brev*, *Brevibacillus*; *P.elg*, *Paenibacillus elgii*; *P.put*, *Pseudomonas putida*; *S.hom*, *Staphylococcus hominis*; *E.clo*, *Enterobacter cloacae*

TS14 and TS27 isolates formed a common clade with *P. putida* strain Dn1.2 and *E.cloacae* strain SB3013, respectively, with 100 % similarity (Fig. 1). Isolates TS9, TS26, TS3 and TS15 shared the clades with *B. humi*, *S. hominis*, *Brevibacillus* and *P. elgii*, with 100 % similarity (Fig. 1). TS2, TS8, TS20, TS22 were grouped with *B. pumilus* and isolates TS5, TS13 with *B. subtilis* strains. TS4, TS7, TS11, TS16, TS17, TS18 were grouped with *B. licheniformis* strains (Fig. 1)*. Bacillus* species were obtained as prominent bacterial endophytes in the present study (Tables 1, 3; Fig. 2) which is supported by earlier reports that *Bacillus* strains are frequently obtained as endophytes following isolation by culture dependent approach.

**Table 3:** Colonization frequency of culturable endophytic bacterial isolates of saffron

Endophytic bacteria	Phylum	Corm (N = 126)	CF-1 (%)	Leaf (N = 126)	CF-2 (%)	Total (corm + leaf) (N = 252)	CF-3 (%)	Dominance (%)
*Bacillus licheniformis*	Firmicutes	4	3.17	11	8.73	15	5.95	27.78
*Bacillus pumilus*		3	2.38	7	5.55	10	3.97	18.53
*Bacillus cereus*		0	0	7	5.55	7	2.78	12.98
*Bacillus subtilis*		0	0	4	3.17	4	1.59	7.42
*Bacillus humi*		0	0	2	1.59	2	0.79	3.69
*Bacillus safensis*		2	1.59	0	0	2	0.79	3.69
*Brevibacillus* sp.		0	0	2	1.59	2	0.79	3.69
*Pseudomonas putida*		0	0	4	3.17	4	1.59	7.42
*Paenibacillus elgii*		0	0	4	3.17	4	1.59	7.42
*Staphylococcus hominis*		2	1.59	0	0	2	0.79	3.69
*Enterobacter cloacae*	Gamma-Proteobacteria	2	1.59	0	0	2	0.79	3.69

CF-1: Colonization Frequency of endophytes in saffron corms; CF-2: in saffron leaves and CF-3: in saffron plant (corm + leaves), N = Number of segments incubated

*Bacillus licheniformis* was found to be the dominant bacterial endophyte of saffron with dominance value of 27.78 % followed by *B. pumilus* with the value 18.53 % (Table 3). Colonisation frequency of dominant endophytes was more in leaf segments than the corm segments of saffron (Table 3). However, Naik et al. (2009) have reported dominant endophytes with more colonisation frequency in roots than in leaf segments of rice. The difference in colonisation frequency of bacterial endophytes among different tissue segments in the present study can be explained on the basis of the fact that colonization frequency of endophytes is tissue dependent as influenced by structure and substrate differences in different tissues of same host plant (Fisher and Petrini 1988; Okane et al. 1998). Isolation frequency of bacterial endophytes from the corm and leaf tissues of saffron indicated presence of *Bacillis licheniformis*, *B. pumilus* and *Paenibacillus* sp. in both the tissues, while *B. safensis*, *Staphylococcus hominis* and Enterobacter cloacae, were restricted only to corm tissue. *Enterobacter* sp. and *Staphylococcus* species have been reported as endophytes of corn, cotton and apple (McInroy and Kloepper 1995; Phukon et al. 2013). On the other hand *B. cereus*, *B. subtilis*, *B. humi*, *Brevibacillus* sp. and *Pseudomonas putida* were restricted to leaf tissue of saffron. Bourgue et al. (2013) also reported *Bacillus subtilis* and *Bacillus pumilus* as endophytes from leaves of switchgrass. These differences reflect tissue specificity of individual endophytic bacterial species which may be the consequence of metabolic ability of endophytic microbes to utilize specific substrate (Carroll and Petrini 1983). Shannon-Wiener index (Table 4) indicated that the diversity of bacterial endophytes was more in leaf tissue (1.94) than in corm tissue (1.54) of saffron. The evenness index was higher in corm tissue and lower in leaf tissue of saffron due to frequent isolations of the same species and rare or no appearance of other isolates in the leaf tissue of saffron (Table 4; Fig. 2). Variation in evenness index accounts for unequal distribution of individuals (Maheswari and Rajagopal 2013).

**Table 4:** Species diversity in terms of dominance and evenness of endophytic bacterial assemblage in different tissue of saffron

Plant part used for endophyte isolation	Total number of species	Total number of isolates	Shannon–Wiener index (H)	Species evenness (E)
Corm	5	13	1.54	0.96
Leaf	8	41	1.94	0.93

**Fig. 3:**
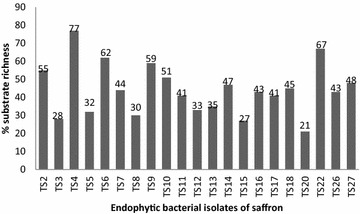
Percent substrate richness of the culturable endophytic bacterial isolates of saffron

**Fig. 4:**
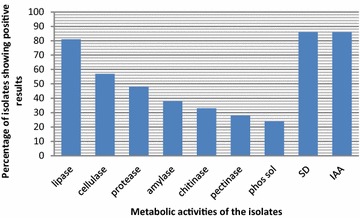
Percentage of isolates showing different metabolic activities; *P+* phosphate solublisation, *SD+* siderophore production, *Iaa+* indole acetic acid production

Percent substrate richness values of different isolates based on carbon source utilization results (BIOLOG) are shown in Fig. 3. These values represent the metabolic potential of the isolates and the variation in the substrate richness values depicts the functional diversity of the isolates. *Bacillus licheniformis* strain TS4 was found to be metabolically most active among all the isolates with substrate richness value of 77 % (Figs. 3, 4). Amylase, cellulase, protease and lipase enzyme production potential of microbes is related to their host plant colonization ability and nutrition, whereas chitinase and phosphate solubilization, siderophore and phytohormone production potential is related to host plant growth promotion. Seventeen isolates were found to be positive for lipase activity, twelve isolates for cellulase activity, ten for protease activity, eight for amylase, seven for chitinase and six isolates showed pectinase activity (Table 1; Fig. 4). Eighteen isolates were found to be positive for siderophore production and IAA production each (Table 1; Fig. 4). As already reported by other workers, saffron plants contain pectins in high amount (Katariya et al. 2011). The pectin solublizing endophytes might be metabolizing host pectins for their nutrition and inturn may be producing some useful metabolites for the host plant. Another significant report implies that endophytes capable of degrading pectic substances are more likely to be latent pathogens of the host plant (Choi et al. 2005). So, pectin solublising bacterial isolates seem to be the latent pathogens of saffron. Five isolates were found to solublize tricalcium phosphate in Pikovskayas agar (Table 1; Fig. 4). The results are in accordance with the report of in vitro solubilisation of inorganic phosphate by endophytic bacteria from rice varieties (Duangpaeng et al. 2013). Chitinase producing isolates might be helpful in plant defense against fungal pathogens.

Isolate TS13, identified as *Bacillus subtilis* was found to be positive for plant growth promoting activities like siderophore and indole acetic acid production. *B. subtilis* has already been reported to possess saffron plant growth promotion potential. According to Eldin et al. (2008) saffron corms treated with *B. subtilis* suspension result in speeding of corm growth, increased stigma biomass as well as increased carotenoid pigment production. Liu et al. (2009) reported that the ginseng seedlings exhibit increased biocontrol, growth promotion and stress resistance properties when sown in soil inoculated with *B. subtilis. B. licheniformis* strains TS4, 7, 11,16 and 17 have shown phytohormone production potential. Isolate TS-2 a *B. pumilus* strain was screened as an agent with maximum enzyme production potential (Table 1). *B. pumilus* and *B. licheniformis* isolated from the rhizosphere of *Alnus glutinosa* have been reported to produce physiologically active gibberllins having role in plant growth promotion (Manero et al. 2001). *B. licheniformis* has also been reported to possess antagonistic potential against tomato and strawberry gray mould, *Botrytis cinerea* (Lee et al. 2006; Kim et al. 2007). *Paenibacillus elgii* strain TS15 was found to be positive for all the plant growth promotion activities tested in the present study. *Paenibacillus* isolated from the rhizosphere of *Calendula* sp. has been reported to possess biocontrol and plant growth promotion potential (Ryu et al. 2005; Kaki et al. 2013). *B. safensis* strain TS22 and *B. humi* strain TS9 were found to be postive for cellulase, siderophore as well as IAA production. To the best of our knowledge, *Bacillus humi* and *B*. *safensis* have not been reported as endophytes from any plant so far.

Identification results for most of the endophytic bacterial isolates (80 %) obtained from BIOLOG coincide with those based on 16S rDNA sequences, further confirming the identification of the isolates (Table 1). Hung and Annapurna (2004) also used BIOLOG for the identification of endophytic bacterial isolates from nodules of soyabean. Isolates identified as same species on the basis of molecular characterisation (Fig. 1) differed in their carbon source utilization profile (Fig. 3), enzyme production profile and antifungal potential (Table 1; Fig. 4). Therefore, these isolates can be considered as different strains of the, however, further studies need to be undertaken to confirm. Different *Bacillus* spp. reported in the present study as endophytes in saffron plant include *Bacillus licheniformis*, *B. subtilis*, *B. pumilus* and *B. cereus*, *Brevibacillus* sp., *B. humi*, *Paenibacillus* sp. and *B. safensis* (Tables 1, 3) (Figs. 1, 2). *Bacillus* species have been reported as potential plant growth promoters as well as biocontrol agents against different plant pathogens (Dasgupta et al. 2006; Gheorghe et al. 2008). Ryu et al. (2005) used *Bacillus* species viz. *Bacillus pumilus*, *Bacillus subtilis* and *Paenibacillus* for growth promotion of *Arabidopsis* sp. Predominance of *Bacillus* species can be attributed to their multienzyme as well as antibiotic production potential. *Bacillus* species have been reported to be potential antagonists of different plant pathogens due to their ability to produce a broad variety of antibiotics (Dikin et al. 2006). Ahmed et al. (2007) have also reported *Bacillus* sp. to suppress root rot disease caused by *F. oxysporum*, causal agent of saffron corm rot. Chitinolytic potential of the isolates might be responsible for their antifungal activity (Devkota et al. 2011). Results indicate that most of the isolates possess plant growth promotion traits and may have important role in biology of saffron by promoting plant growth directly, indirectly or synergistically.

## Conclusion

The present study reports presence of morphologically and metabolically diverse endophytic bacterial assemblage in *Crocus sativus* (saffron). Endophytic bacteria have been isolated from corm (belowground) and leaf (aboveground) tissue of saffron. It has been observed that multiple plant tissue sampling, polyphasic characterisation and mathematical analysis of data are helpful in determining the endophytic biodiversity in a given plant species. However, more tissue sampling is required to uncover the complete culturable bacterial endophytic microbiome of saffron. Moreover, minimal media can also be used for the isolation of slow growing bacterial isolates, if any. The metabolic analysis of the isolates demonstrated that they produce number of hydrolytic enzymes which might play important role in nutrient cycling. The plant growth promoting properties indicate their potential as biotechnologically important cultures. Further studies are required to exactly determine the role of the endophytic isolates in the biology of saffron plant. Potential isolates from the present repository of saffron endophytes can be used for field trials to confirm the feasibility of using the same in plant growth promotion and disease management programmes. Such studies would facilitate the selection of promising cultures possessing desirable host plant growth promotion properties and can be exploited as biofertilizers in saffron fields. The use of microbial agents are becoming indispensable alternative to chemical fertilizers. So this would surely be a step forward towards sustainable and developed agriculture by minimising the use of chemicals in the environment.

## Methods

### Collection of the plant material

Symptomless corms were collected from saffron fields of Pampore, Kashmir (34.02°N; 74.93°E; 5164 feet high), India, in August 2013 (dormant stage of the saffron plant). Collected samples were brought to laboratory under low temperature conditions and processed for isolation of endophytes. Healthy corms were used for the direct isolation of bacterial endophytes whereas some of them were sown in the garden soil. Leaves were collected in November 2013 (during vegetative phase) and used for the isolation of bacterial endophytes.

### Isolation of endophytes

The corms and leaves were surface sterilized by following the protocol of Santos et al. (2003) with slight modifications. Sterilization involved washing of selected plant parts in running water for 10 min, washing twice in distilled water for 1 min followed by treatment with 70 % (v/v) ethanol for 1 min. Subsequently, the explants were treated with 0.5 % (v/v) sodium hypochlorite for 30 s and again treated with 70 % ethanol for 1 min. Each step was followed by washing with sterile water for 2 min. Surface sterilized plant parts were later dried on sterile filter paper. After proper sterilization, leaves and corms of saffron were cut into small segments (0.5–1 cm). The segments were placed on three different media plates, viz. Nutrient agar, Potato dextrose agar and 1 % Water agar, supplemented with 50 µg/ml cycloheximide (Verma et al. 2009), for the isolation of bacterial endophytes. Effectiveness of surface sterilization was validated by following the protocol of Verma et al. (2009) with slight modifications. Surface sterilized tissue segments were stirred in sterile water and 500 µl of the suspension was inoculated on nutrient agar plates. As another control, surface sterilized explants were touched on the media plates and observed for the growth of surface bacteria, if any. All the inoculated plates were incubated at 37 °C and monitored at regular intervals for the growth. Endophytes thus observed growing on explants were isolated, purified and stored as slants at 4 °C till further use.

### Screening of bacterial endophytes

Morphologically and biochemically different isolates were selected for further study. Morphological features studied included colony texture, margins, colour, elevation, growth, form, shape of bacterial cells (rod, cocci or coccobacillus) and Gram’s staining reaction (Steinbach and Shetty 2001). Biochemical characterisation was done using Bacterial identification kit (HiMedia KB003) as per user manual instructions (Results available as supplementary data).

### Phylogenetic diversity analysis

Genomic DNA was isolated from the selected bacterial cultures using Himedia bacterial DNA purification kit as per manufacturer’s instructions. Universal bacterial primers were used for the amplification of 16S rRNA gene viz 16Bacf- 5′AGAGTTTGATCCTGGCTCAG 3′ (forward primer) and U1492R- 5′ GGTTACCTTGTTACGACTT 3′(reverse primer) (Doty et al. 2005). PCR program included initial denaturation at 94 °C for 4 min followed by 30 cycles of denaturation at 94 °C for 1 min; annealing at 54 °C for 50 s, extension at 72 °C for 2 min and final extension at 72 °C for 10 min.

The resulting PCR products were purified by gel extraction using DNA purification kit (Axygen) as per manufacturer’s protocol. The purified 16S rDNA amplicons were sequenced by Sanger’s method at SciGenom labs Chennai, India. The obtained sequences were then compared with public databases at the NCBI site by using the BLASTn algorithm (Altschul et al. 1997). Sequences were submitted to GenBank and accession numbers were obtained. Phylogenetic and molecular evolutionary analysis were conducted using MEGA version 6 (Tamura et al. 2013). The evolutionary history was inferred using the UPGMA method (Sneath and Sokal 1973). The evolutionary distances were computed using the Kimura 2-parameter method (Kimura 1980) and are in the units of the number of base substitutions per site.

### Screening of metabolic diversity

The selected isolates were analysed for different metabolic activities including carbon source utilization ability, enzyme production potential viz lipase, amylase chitinase, cellulase, protease and pectinase as well as plant growth promoting properties like phosphate solubilisation, siderophore production, indole acetic acid production. Determination of functional diversity in terms of carbon source utilization ability of the isolates and their subsequent species level identification was carried out by using BIOLOG identification system with OMNILOG version 2.3.01; reader version Rev1.07 and GEN III database (Garland and Mills 1991; Miller and Rhoden 1991; Amaresan et al. 2012). A similarity index value of more than or equal to 0.5 was considered to indicate good species match (Wu et al. 2013). Percent substrate richness value was calculated for each bacterial isolate by determining the number of carbon compounds utilized per microplate (Miglia et al. 2007). Plate assays were done to assess the enzyme production potential of the isolates for amylase (starch agar); lipase (tributyrin agar); pectinase (MP-5 agar); protease (0.5 % gelatine agar); cellulose (1 % carboxymethylcellulose agar); chitinase (0.7 % chitin agar) (Kumar et al. 2012). Different enzyme media were inoculated with the bacterial isolates and observed after 48 h incubation at 37 °C. Phosphate solubilization potential of the isolates was assessed on Pikovskayas agar (Kumar et al. 2012). Saffron endophytes were analysed for phytohormone IAA production by following the method of Gordon and Weber (1951). Siderophore production was detected by the formation of orange halos on chrome azurol S agar (CAS) agar plates after 48 h incubation at 37 °C following the protocol of Alexander and Zuberer (1991).

On the basis of data on morphological, molecular and biochemical characterization, repetitive isolation of same bacterial endophytes was determined and isolation rate, colonization frequency, isolation frequency and dominance values were calculated.

Percent Isolation rate (IR) of bacterial endophytes was calculated as (Maheswari and Rajagopal 2013):

$\frac{NI}{Nt} \times 100$ where NI is number of endophytic isolates obtained from plant segments and Nt is total number of segments incubated (Nt).

Percent colonization frequency (CF) of endophytes was calculated as (Maheswari and Rajagopal 2013):

$\frac{Ncol}{Nt} \times 100$ where Ncol is number of plant segments colonised by single endophyte.

The dominance of endophytes was calculated (Verma et al. 2007) as:

$\frac{CF}{{\sum {CF} }}$ where CF is the percentage colonization frequency of a given endophyte and ∑CF is the sum of the percentage colony frequencies of all the endophytes.

Percent isolation frequency (IF) of the endophytes was calculated as (Maheswari and Rajagopal 2013):

$\frac{N1}{N2} \times 100$ where N1 is number of times single isolate obtained and N2 is total number of isolates obtained.

For analysis of endophytic bacterial diversity in saffron plant tissues Shanon diversity index (H) and Species eveness (E) values were determined (Verma et al. 2007).

## Additional file

10.1186/s40064-015-1435-3 Morphological and biochemical characterisation of bacterial endophytes isolated from leaf and corm of saffron.
