# Supplementary material for: Diversity of culturable bacterial endophytes of saffron in Kashmir, India
Source: Springerplus. 2015 Nov 2;4:661. doi: 10.1186/s40064-015-1435-3 (PMC4630316; doi:10.1186/s40064-015-1435-3)
Supplement: Supplementary file 1 — 10.1186/s40064-015-1435-3 Morphological and biochemical characterisation of bacterial endophytes isolated from leaf and corm of saffron. [file 40064_2015_1435_MOESM1_ESM.pdf]

Table-S1 Morphological features of bacterial endophytes isolated from leaf and corm of saffron

Endophytic bacterial isolates	Colony Characteristics				
	**Texture**	**Margin**	**Colour**	**Elevation**	**Growth**
TS-2	Smooth and sticky	Wavy	Yellowish white	Raised	Fast
TS-3	Smooth and sticky	Entire	Transparent	Flat	Slow
TS-4	Rough and dry	Entire	Dirty white	Raised	Moderate
TS-5	Smooth and sticky shiny	Filamentous	Yellowish white	Flat	Fast
TS-6	Smooth and sticky	Wavy	Dirty white	Flat	Fast
TS-7	Rough and dry	Filamentous	Dirty white	Flat	Fast
TS-8	Smooth and sticky	Entire	Light yellow	Raised	Moderate
TS-9	Smooth and sticky	Wavy	Light yellow	Raised	Moderate
TS-10	Smooth and dry	Wavy	Light yellow	Flat	Fast
TS-11	Rough and dry	Entire	Dirty white	Raised	Moderate
TS-12	Smooth and sticky	Filamentous	Dirty white	Flat	Fast
TS-13	Rough and dry	Filamentous	yellowish white	Flat	Moderate
TS-14	Smooth,sticky and shiny	Filamentous	Light yellow	Flat	Moderate
TS-15	Smooth and sticky	Entire	White	Raised	Slow
TS-16	Rough and dry	Filamentous	Dirty white	Flat	Fast
TS-17	Rough and dry	Filamentous	White	Flat	Fast
TS-18	Smooth and sticky	Entire	Dirty white	Raised	Moderate

TS-20	Smooth and sticky	Entire	White	Raised	Fast
TS-22	Smooth, sticky and shiny	Entire	White	Smooth	Fast
TS-26	Smooth,sticky and shiny	Entire	Dirty white	Raised	Moderate
TS-27	Smooth and sticky	Entire	Light yellow	Raised	Fast

Table-S2: Biochemical characterisation of endophytic bacterial isolates of saffron based on substrate utilisation

**Biochemical Test**	Endophytic bacterial isolates (TS)																				
	2	3	4	5	6	7	8	9	10	11	12	13	14	15	16	17	18	20	22	26	27
ONPG	-	-	+	-	-	+	-	-	+	+	-	-	+	-	+	+	+	-	-	-	-
Lysine utilization	-	-	-	-	-	-	-	-	-	-	-	-	-	-	-	-	-	-	-	-	-
Ornithine utilization	-	-	+	-	-	-	-	-	-	+	-	-	-	-	-	-	-	-	-	-	-
Urease	-	+	+	-	-	+	-	-	-	+	-	-	-	-	+	+	-	-	-	-	-
Phenylalanine deamination	-	-	-	-	-	-	-	-	-	-	-	-	-	-	-	-	-	-	-	-	-
Nitrate reduction	-	+	+	+	+	+	-	+	-	+	-	+	-	+	+	+	-	-	-	+	+
H2S production	-	-	-	-	-	-	-	-	-	-	-	-	-	-	-	-	-	-	-	-	-
Citrate utilization	+	+	+	+	+	+	+	+	+	+	+	+	+	+	+	+	+	+	+	-	+
Voges Proskauer’s	+	-	+	+	-	+	+	-	+	+	-	+	+	-	+	+	+	+	+	-	+
Methyl red	-	-	-	-	-	+	-	-	-	-	-	-	-	-	-	-	-	-	-	-	+
Indole	-	-	-	-	-	-	-	-	-	-	-	-	-	-	-	-	-	-	-	-	-

Malonate utilization	+	-	-	+	+	+	+	-	+	+	+	-	+	-	+	+	+	+	-	-	+
Esculin hydrolysis	+	-	+	+	-	+	+	+	+	+	-	+	+	-	+	+	+	+	+	-	+
Arabinose	-	-	+	-	-	-	-	-	-	-	-	-	-	-	-	+	-	-	-	-	-
Xylose	-	-	-	-	-	-	-	-	-	-	-	-	-	+	-	-	-	-	+	-	+
Adonitol	-	-	-	-	-	-	-	-	-	-	-	-	-	-	-	-	-	-	-	-	-
Rhamnose	-	-	-	-	-	-	-	-	-	-	-	-	-	-	-	-	-	-	-	-	-
Cellobiose	-	-	+	+	-	+	+	-	+	+	-	+	-	-	-	+	-	-	+	-	+
Melibiose	-	-	-	-	-	-	-	-	-	-	-	-	-	+	-	-	-	-	+	-	+
Saccharose	-	-	+	+	-	+	+	+	+	+	+	+	-	+	-	+	-	+	+	+	+
Raffinose	-	-	-	-	-	-	-	-	-	-	-	-	-	-	-	-	-	-	-	-	+
Trehalose	-	-	+	+	+	+	+	+	+	+	+	+	-	+	-	+	-	+	+	-	+
Glucose	+	+	+	+	+	+	+	+	+	+	+	+	+	+	+	+	+	+	+	+	+
Lactose	-	-	-	-	-	-	-	-	-	-	-	-	-	-	-	-	-	-	-	+	-
Oxidase	+	+	+	+	+	+	+	+	+	+	+	+	+	+	+	+	+	+	+	-	+

'+' indicates bacterial isolate showing positive test for the substrate whereas '-' sign indicates negative test
